# Assessing Acceptability, Feasibility, and Preliminary Effectiveness of a Community-Based Participatory Research Curriculum for Community Members: A Contribution to the Development of a Community–Academia Research Partnership

**DOI:** 10.1089/heq.2018.0034

**Published:** 2018-10-09

**Authors:** Julio C. Jiménez-Chávez, Fernando J. Rosario-Maldonado, Jeremy A. Torres, Axel Ramos-Lucca, Eida M. Castro-Figueroa, Lydia Santiago

**Affiliations:** ^1^ School of Behavioral and Brain Sciences, Ponce Health Sciences University, Ponce, Puerto Rico.; ^2^ Public Health Program, Ponce Health Sciences University, Ponce, Puerto Rico.; ^3^ Public Health Program, University of Puerto Rico, Río Piedras, Puerto Rico.

**Keywords:** CBPR curriculum, community capacity building, community empowerment

## Abstract

**Purpose:** The community-based participatory research approach has been identified as a great asset in reducing health disparities through the integration of community members in all phases of the research process. It is essential to provide skills to community members to achieve successful research partnerships. The purpose of this study is to evaluate the feasibility, acceptability, and preliminary efficacy of the community-based participatory research training curriculum for community members.

**Methods:** Using mixed-methods, noncomparative design, eight workshops were developed and tested. Workshops covered topics such as community-based participatory research principles, health disparities, ethics in community-based participatory research, and fundamentals of research methodology. A total of 25 community leaders were trained. Pre-/post-test knowledge (unpaired *t*-test), retention rate, workshop satisfaction, and cognitive debriefing sessions were used to assess knowledge gained and acceptability and feasibility of the curriculum.

**Results:** A retention rate of 100% and an average satisfaction of 92.68% were obtained. Preliminary effectiveness results indicate that there was an overall significant change in participant's knowledge before and after the curriculum (*p*<0.001). In the cognitive debriefing, participants were satisfied with the organization and structure and found the curriculum feasible. Furthermore, participants identified the skills acquired to aid in being more effective in their communities and work with academic researchers. The following changes were recommended: workshops' order, time, practical activities, and level of language.

**Discussion:** Findings from this study suggest that the curriculum was acceptable and feasible to community leaders and that it might provide skills to actively incorporate community members in research activities. A large randomized clinical trial (RCT) study to evaluate curriculum effectiveness is recommended.

## Introduction

Hacker et al. defined community capacity building as “the cultivation and use of transferable knowledge, skills, systems, and resources that affect community- and individual-level changes consistent with public health-related goals and objectives.”^1^ It is an essential component of and a guiding principle in community-based participatory research, as it fosters colearning among all partners^2–4^; its importance revolves around the notion that capacity building leads to an equal sharing of power in the researcher–community member relationship.^5,6^ Furthermore, community capacity has been identified as “the characteristics of communities that affect their ability to identify, mobilize, and address social and public health problems.”^7,8^

The capacity building of community stakeholders in community-based participatory research is to the mutual benefit of both partners, as it empowers community coinvestigators to foster greater collaboration and the shared ownership of research achievements and gives equal power in the dyadic relationship. Furthermore, it supports existing and potential community-based participatory research projects.^9^ The findings of a systematic review underscore the importance of building capacity to increase research literacy and of sharing knowledge in general with community members.^10^ This same review, which explored the effectiveness of clinical trials that used community-based participatory research methodology involving racial and ethnic minorities, revealed that community partners most frequently were involved in participant recruitment and the development and delivery of interventions.^10^ Furthermore, these community partners participated in the interpretation of quantitative research only 21% of the time and in the dissemination of research findings only 47% of the time.^10^ These results could be attributed to differences in research knowledge, limited resources, and the capacity of academic partners to teach community partners the necessary skills to participate fully in the stages of research.^10–14^

Little work has been done in pinpointing ways to reduce barriers for community members to fully participate in community-based participatory research,^11,15^ which is very important, if what has been previously stated to be of benefit to both the community members and academics is to be achieved. Although curriculums/programs have been created to teach community members about community-based participatory research and/or health disparities,^5,11,16–20^ to our knowledge, a curriculum that would provide Hispanic community leaders with a basic understanding of community-based participatory research, research, and health disparities has not been developed. Recent curriculum topics center on public health components, community-based participatory research components, or research components but do not unite all of these components together in a single curriculum.^5,11,16–20^ We identify community leaders, as defined by Community Tool Box, as people who take responsibility for the well-being and actively participate in the improvement of their community.^21,22^

To address the existing knowledge gaps, a curriculum (centered on health disparities) to train Hispanic community leaders in community-based participatory research principles and basic research concepts was created. The primary goal of the community-based participatory research training for community leaders was to develop an innovative curriculum aimed at forming a new generation of community leaders skilled at engaging in community/academic research partnerships for the elimination of health disparities in our communities. The purpose of this article is to report on the development and creation of a curriculum and evaluate not only its feasibility and acceptability but also the knowledge gained (preliminary effectiveness) by the community leaders.

## Methodology

### Study design and procedure

The researchers conducted a mixed-methods pilot study to assess a community-based participatory research curriculum for training community members. The Institutional Review Board approved the study protocol at the Ponce Health Sciences University.

### Curriculum design

A review of the literature showed common themes regarding community-based participatory research principles as they apply to research. Also, themes included in the *Inter-Professional Perspectives of Health Disparity* course^23^ at Ponce Health Sciences University were considered. Finally, the topics that were selected to comprise the curriculum were presented to three experienced researchers in the community-based participatory research approach, which suggested the six topics in Table 1.

**Table 1.** **Workshop Topics and Their Respective Objectives**

Workshop topics	Objectives
CBPR (two sessions)	Provides scientific information about the basics of CBPR Highlights the benefits of including community members as partners in the research process
FRM (two sessions)	Provide the basic concepts of research methodology to facilitate the understanding of all phases of the research process Describe the different ways in which the community can contribute to research
Work ethics in CBPR	Creates awareness about the specific ethical aspects to be considered when adopting CBPR practices
HD	Develop a conceptual foundation to understand the role of health disparities in health warfare, along with the implications of such disparities in terms of research and health outcomes
TR	Presents an updated review of the translational research concept, the relevance of this concept in terms of basic and social science research, knowledge transference, and the contribution of community members in translational research projects Presents the processes involved in translational research projects and in leading multidisciplinary teams that include community members, among others
SDH	Demonstrate how social conditions, economic factors, and the political and social structures in which people live are related to the distribution of power, money, and resources and how these factors impact the epidemiology of medical conditions

CBPR, community-based participatory research; FRM, fundamentals of research methodology; HD, health disparities; SDH, social determinants of health; TR, translational research.

Experts on the topics and who had extensive academic/community experience were recruited to give the workshops. They were given a rubric to establish uniformity between the workshops, which included assigned topics, objectives of the workshop (as well as its duration, format, and content), practical activities, and questions to be used to evaluate knowledge gained (including the number of questions and the issues to be considered when writing those questions). Before being presented, workshops were revised to ensure the suitability of the established objectives.

### Participant recruitment procedure

Purposive sampling was utilized to recruit 25 community leaders from the “Programa de Comunidades Especiales”^24^ (*Special Communities Program,* in English), which is a government-based initiative that seeks to empower underserved and socioeconomically disadvantaged communities in Puerto Rico as a way to promote self-management. Participants in the study represented various communities and community-based organizations (linked to social or health interests) on the south side of Puerto Rico and were active community leaders.

**FIG. 1.**
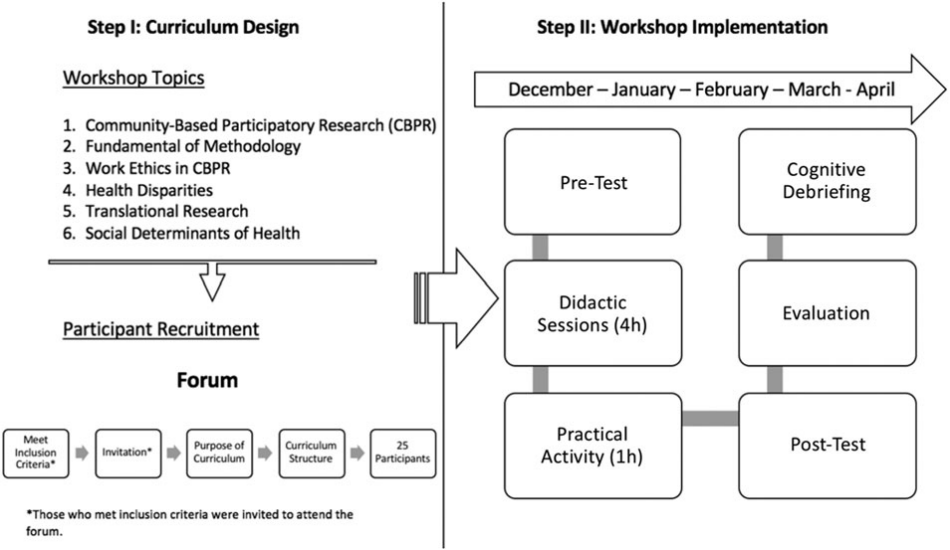
Flowchart of curriculum design, participant recruitment, and workshop implementation. *Those who met inclusion criteria were invited to attend the forum.

A community forum was held to recruit leaders from the *Special Communities Program*; there, we presented the goal, objectives, and purpose of the project. An invitation letter to the forum was sent to 40 community members that met inclusion criteria: (a) be 21 years or older, (b) have completed at least a high school education, (c) voluntarily have consented to participate in the workshop training, (d) be a designated community leader of the *Special Communities Program* southern side, (e) express interest in learning about community-based participatory research activities, (f) express interest in becoming a community research leader, and (g) express availability/commitment to attend all the workshops and complete the cognitive debriefing tool. After presenting the community-based participatory research curriculum objectives and activities, we invited community leaders to participate in it, see Figure 1. The participants who enrolled signed informed consent after discussing the document, completed a sociodemographic questionnaire, and were given a calendar with the schedule of the workshops. Participants received $100, per workshop, for their time/effort, transportation, and meals.

### Workshop implementation

The curriculum consisted of eight workshops, facilitated by academics with broad knowledge of community-based participatory research-related topics. All the workshops consisted of interactive group sessions, didactic session (4 h), and practical activity (1 h), which included the following topics: community-based participatory research (two sessions), fundamentals of research methodology (two sessions), work ethics in community-based participatory research, health disparities, translational research, and social determinants of health. Workshops were held from December 2016 to April 2017 (one or two sessions/month) at Ponce Health Sciences University.

#### Measures

##### Qualitative data (acceptability and feasibility)

To assess the acceptability and feasibility of the curriculum, the participants completed a cognitive debriefing session at the end of every workshop. Each session included questions exploring general thoughts, content, confusion of content, speaker style, controversial topics, practical activity, and readiness to educate the community.

- Debriefing (workshops, group discussions)
  Cognitive debriefing is the process by which an instrument is actively tested with representatives of the target population and target language group to determine if the respondents understand the material.^25,26^ In this study, it was utilized to evaluate the feasibility and acceptability of the curriculum and the topics presented to the participants and to improve the quality of the workshops. At the end of every workshop and implementation period, the participants had a cognitive debriefing led by a trained interviewer. The final cognitive debriefing at the end of the implementation period was done in four smaller groups with five to six participants and was centered more specifically on the curriculum as a whole. The questions used for the cognitive debriefing were designed in a semistructured format, and each session lasted from 30 to 60 min, depending on how actively the members participated. Suggestions from the cognitive debriefing sessions after the workshops were reviewed and utilized for later workshops.

Recordings were made of every group session, so they later could be transcribed. Two research assistants independently reviewed and analyzed the contents of the recordings. Themes and responses that were repeated during the various workshops and at the end of the implementation period were noted. After independently reviewing and analyzing the contents, researcher assistants categorized those contents, and any conflicting codes that were found were discussed by the two research assistants and the principal investigator. The principal investigator decided on the final coding after this discussion.

##### Quantitative data (acceptance and knowledge gained).

- Retention rate
  High retention rates are important to maintain the internal validity of a study, especially when working with a cohort.^27^ In this study, the term “retention” refers to the completion of six or more workshops by the participants and “dropout” to describe participants who decided to discontinue workshops and/or who completed five workshops or fewer. The retention rate formula is as follows:
  \begin{align*}
\left( {{ \rm{retained}}} \right) / \left( \textbf{\textit{n}} \right) \times \;100
\end{align*}
- Satisfaction
  The participants, at the end of each workshop, evaluated that specific workshop for satisfaction through an evaluation form with a 5-point Likert scale. The two highest points were consolidated and considered satisfactory.
- Knowledge gained (preliminary effectiveness)
  Before and after the didactic session, participants were administered pre- and post-tests to evaluate the knowledge acquired from the workshops. Each lecturer created between 5 and 10 multiple choice questions using, as reference, the information presented in each of the workshops; the questions were presented to and approved by the principal investigator to verify comprehensibility of language and fidelity of rubric. IBM SPSS Statistics (version 24) was utilized to assess the change from pretest to post-test using unpaired *t*-test analysis. The pretest and post-test were assigned group 1 and 2, respectively, for the analysis.

## Results

### Sociodemographic

Table 2 shows sociodemographic results. Female participants represented 68% of the sample, and more than half were from 45 to 64 years old (56%). Twenty-four percent of the participants had a monthly income of $500 or less, and 56% were married, and highest educational degree obtained was a bachelor's degree (10%).

**Table 2.** **Sociodemographic Characteristics of Participants**

Variable	*n*	%	Variable	*n*	%
Sex			Monthly income		
Male	8	32	$500 or less	6	24
Female	17	68	$501–$1,000	2	8
Total	25	100	$1,001–$1,500	2	8
Age, years			$1,501–$2,000	3	12
25–34	4	16	$2,001–$2,500	4	16
35–44	3	12	$2,501–$3,000	3	12
45–54	9	36	$3,001 or more	5	20
55–64	5	20			
65 or older	4	16			
Total	25	100	Total	25	100
Marital status			Education		
Single/never married	5	20	Completed HS	5	20
Married	14	56	Technical degree	3	12
Cohabiting with partner	2	8	Associate degree	6	24
Divorced	4	16	Bachelor's degree	10	40
			Doctorate degree	1	4
Total	25	100	Total	25	100

#### Quantitative results

#####  Acceptance

- Retention rate
  During the implementation, no dropouts were evidenced resulting in a retention rate of 100%.
- Satisfaction
  Workshops were given a more than 85% satisfaction rate, and individual rates of the workshops are as follows: community-based participatory research (S1: 92%, S2: 91%), fundamentals of research methodology (S1: 96%, S2: 88%), work ethics in community-based participatory research (96%), health disparities (100%), translational research (86%), and social determinants of health (92%). The overall satisfaction of the workshops was estimated at 93%.
- Cognitive debriefing
  See Table 4 for cognitive debriefing results of acceptance.

##### Knowledge gained

Unpaired *t*-test results indicate that there was a change in participants' knowledge after four of the seven workshops in which a pre-/post-test was administered as seen in Table 3.

**Table 3.** **Unpaired *t*-Test Results of the Change in Knowledge in Participants (Measure of Preliminary Effectiveness)**

Workshop topics	*n*	MD	SED	95% CI	*t*	Sig. (two tailed)
CBPR (two sessions)	S1: 25	^a^	^a^	^a^	^a^	^a^
	S2: 23	S2: 0.39	S2: 0.34	S2: −0.30 to 1.08	S2: 1.14	S2: 0.26
FRM (two sessions)	S1: 24	S1: 0.12	S1: 0.29	S1: −0.45 to 0.70	S1: 0.43	S1: 0.67
	S2: 25	S2: 0.56	S2: 0.34	S2: −0.12 to 1.24	S2: 1.65	S2: 0.11
Work ethics in CBPR	24	2.08	0.42	1.23–2.93	4.93	0.00
HD	23	3.26	0.51	2.23–4.29	6.38	0.00
TR	24	3.16	0.45	2.26–4.07	7.05	0.00
SDH	25	1.68	0.26	1.16–2.20	6.48	0.00
Overall knowledge gained	168	1.60	0.20	1.20–2.00	7.91	0.00

^a^ Data not available.

95% CI, 95% confidence interval; MD, mean difference; *n*, number of participants who attended and took pre-/post-tests; SED, standard error difference.

#### Qualitative results

##### Feasibility

- Cognitive debriefing
- In the cognitive debriefing sessions, acceptance and feasibility were evaluated using guided questions. Table 4 results were taken from the final cognitive debriefing in which the questions were explicitly centered on the curriculum.

**Table 4.** **Perceived Acceptance and Feasibility with Resource Utility Reported by Workshop Participants Through Cognitive Debriefing Responses**

Topic evaluated/question	Thematic dominions	Responses
Acceptance: What is your general opinion about the training offered by the CBPR program? What can be bettered? How prepared do you feel to collaborate on a research project as a community investigator? Why do you feel prepared or why not? What do you think about the sequence (order) in which the workshops were given? Would you change the order? To what? Which workshop would you place first and why? What is your opinion of the materials that were distributed in each of the workshops? Were they useful? Why or why not? What other materials or resources do you need to better understand the subject matter?		
Difficulty level of the language	Highly technical language	The language is sometimes too technical and may not have been well understood. Along with the short time of the workshop, language could be a factor… There are things that could be taken advantage of a little bit more… Very technical language. Sometimes very technical.
Structure	Training is well organized and structured	I think they have a solid base, plus all the material they gave us was adequate. I think that it was very good and very organized, too. Very well structured.
Division of the workshop sessions	Some of the workshops need to be divided into two or more sessions	Add more time? Maybe no more time but split the material (workshops). More time for the methodology workshop.
Utility	Training provides tools to enhance leaders' functions in their communities and to aid them to work closely with academic researchers All the information is relevant and pertinent	I think that this project allows us to acquire tools so that we can be more effective in what we are doing in our communities, but it also creates in us—well, in my case, it creates an expectation. Because wherever I go, I talk about this, and then my vision is that whatever we are learning here or that everything I have learned here, I am going to use to benefit what I am already doing in the community. Sure! When the researcher talks to us we already know what he is going to talk about. In my case, with all this knowledge that we received, I feel that, yes! [referring to the usefulness of the training] I have transmitted information to others, and I have already taken the step of asking about diseases and am better evaluating the community for when we pull together to do the practice so that I will be able to transfer that knowledge to the researcher, in terms of the survey of the community and the diseases that are around at that moment.
Sequence		
Adequate	The order of the workshops was adequate	The order is very good. There was a very good sequence.
Improve	Modify the workshops' order, first addressing socioeconomic topics/methodology should be last workshop	Before starting the research projects, it is recommended that the empowerment of the community, the determinants of health, and other such issues be addressed first and then the last two workshops be dedicated to research. To have the methods fresh in the memory before starting the practice. It's like giving the workshop and doing the practice, you know? (After each workshop do a practice session/laboratory.) I feel that the penultimate one we took should close the workshops (referring to the Translational Research section).
Resources		
Adequate	Useful materials (hard copies of the PowerPoint presentations and scientific articles)	Excellent! Very good…very helpful.
Improve	Include other methods of providing information: technology (Internet web pages, pen drives) Green perspective	We can have the presentations in PowerPoint and put them on pen drives or access them via a link, and so on. I am more technological, and in the world that we live in, maybe send the material through the Internet because it makes it easier for them and for me, too. Do not hand out paper copies of the presentations… economize, be ecofriendly.
Speakers	Speakers trained and with broad experience on the topics	In all the speakers, we noted high self-confidence, and it showed in the ability with which they spoke… It was good because they all had passed through the experience (of working with the community) and had personal experience with what they were talking about… I think the important thing about their capacity, really, is the experience of the speakers. The speakers' experience should go hand in hand with the topics they are going to talk about.
Feasibility: What is your opinion about the capacity of the program to create community investigators? About its scope? Would you recommend that the program be repeated for other leaders in this and other communities?		
Recommendation		
	The training program should continue Promote information about it among community members	… This is not one more project that is going to be left behind, that we start and then abandon in the long run. We can identify the people in our communities who have the potential to take the workshops, and I am sure they will become allies… All will learn as we did. The leaders in Ponce are very agile minded and would love this type of workshop… This is going to make them useful to their communities; just like all of the others (the participants in the workshop), I loved my classmates' projects, for example, the one about the coal ash… [Speaking of recommending the workshop to others] It is the first time in all of my years working with the community that I have received any training like this; that is, I have been in lots of workshops and they talk about many good topics, but to have them be so precise, so clear, so specific—for me at least, it's the first time.
Improve	Lengthen the total duration of the training and increase the practical activities by topic	If they are going to continue the workshops, they should allot more time. We must continue reading about the topics, but as for the classroom, we need more time. I see the program as being quite complete… The only issue I see is the time. Change the program so that it lasts for a full year, along with practice. … Talk about the theory for 1 day and then practice it on the second day, because there is so much information… information, and we are not bringing it to the practice, though we understand most of it. If so—if it were 2 days, then maybe we could do both at the same time (referring to theory and the practice). There was not enough time for the interpretation of some of the workshops.

## Discussion

Results obtained indicate that participants accepted the curriculum, as the retention rate achieved was 100.0%. Furthermore, participants in the evaluation forms at the end of every workshop reported that they were satisfied with the workshops; a 92.7% overall satisfaction rate was obtained. In their answers to the cognitive debriefing questions related to the acceptance of the curriculum, participants expressed the opinion that some of facilitators used language that was too technical to easily absorb when presenting workshop topics, which should be taken into consideration for future workshops. They also suggested that some workshops need to be given more time or be divided into more sessions and suggested, as well, that the order in which they are given be changed. For example, participants pointed to the methodology workshop as being one that would benefit from more time (to aid understanding); they also recommended that the research-related workshops be placed after those dealing with socioeconomic topics, as the latter is more immediately relevant.

The participants found the curriculum to be well organized and structured, and the workshop topics and the materials given were pertinent and useful to the leaders, not only when they worked with the researchers but also when they were out working in their communities. In every workshop, materials were given, such as PowerPoint handouts and scientific articles. These were identified as being useful, although the participants also mentioned that such materials could be sent electronically (via e-mail) so that each could peruse said materials on their personal computers/smartphones. Acceptability and satisfaction indicators demonstrate that the curriculum resonates with the community leaders, as indicated by the results in their evaluations of the workshops, but this resonance can also be attributable, at least partly, to Puerto Rico's socioeconomic reality. The island's health system and economy have experienced challenges and a decline in the last few years,^28,29^ all of which have impacted many communities, including the ones to which our participating leaders belong. Being aware of this, they could see an opportunity to acquire additional tools and utilize the knowledge they gained, as they were taught that community-based participatory research helps create meaningful scientific investigation, which in turn yields science-based knowledge for broader based social action/change that is intended to benefit their communities. This echoes with the Hispanic values of *familismo,* which places high value on family (both nuclear and extended), and *altruismo,* which is the disinterested concern for the well-being of others based on love or caring.^30,31^ We recognize these characteristics as being associated with one the of community-based participatory research principles, which is to recognize the strengths and assets of the community.^3,4^ These values, being characteristic of the Hispanic population, could increase both the impacts of the overall satisfaction and acceptability of the participants with the curriculum. Other training programs demonstrate findings such as ours, in which, as a result of their participation in said programs, additional tools were obtained by members, which aided them to change their communities for the better.^11,12^

The preliminary effectiveness of the curriculum was measured as knowledge gained; out of the seven workshops analyzed, the knowledge acquired by participants in four of them was found to be statistically significant (*p*<0.05). Three workshops were not statistically significant: fundamentals of research methodology (*p*>0.05, S1 and S2) and community-based participatory research (*p*>0.05). Three of the workshops that yielded statistically significant results covered areas in the social sciences (work ethics in community-based participatory research, health disparities, and social determinants of health), and one covered an area in the basic sciences (translational research). The topics encompassing the social sciences appear to be easier to retain, as the material is relatable to the leaders' daily lives. Assimilation theory confirms this, as it states that the acquisition of information from presented learning material requires a meaningful learning set and the demonstration of potentially meaningful material, later necessitating that a given learner's cognitive structure contains relevant anchoring ideas to which the new material can be related.^31,32^ Although translational research is basic science and our participants achieved significant changes in their knowledge, this could be attributed to the applicability of it regarding their being able to use it to help their communities, as the curriculum involved bringing information from laboratories to the community.^31,32^ Any lack of relatability may have affected the retention of topics such as fundamentals of research methodology, which is a research topic that may not be recognized by leaders as a direct vehicle to help the community, and community-based participatory research, which is a research and social sciences topic that presents the integration of community members as part of the research team; in this, the research methods and roles they will acquire during such a process when participating in community-based participatory research are relatively new to community members, which might explain the lack of change in knowledge in the workshop.^31,32^ The utilization of a practice session at the end of every workshop can further explain positive results; the participants commented that these sessions aided them to solidify their understanding of the material, which, we understand, is related to their being able to familiarize themselves with the content. As previously mentioned, workshop topics were based on topics deemed relevant by our sample population, which relatability also aids in their retention of knowledge and is confirmed by the overall knowledge gained (*p*<0.00).^31,32^ Still, taking into consideration all of the recommendations of the participants and the results obtained, a refinement in the structure of the workshops needs to be made. Suggestions with respect to future workshops include lengthening the duration of the workshops (which could be divided into 4 h of theory and 2–3 h of practice sessions), dividing such topics as community-based participatory research and fundamentals of research methodology into at least three to four sessions, respectively, and sending workshop materials electronically to better accommodate the needs of the participants. Our results are like those of a training program whose participants also expressed their desire that the training be extended, or topics spread across more sessions. A strategy utilized by the same program to help participants solidify knowledge was to give homework assignments, which 88% felt were useful and 73% claimed helped them to understand the material^11^; this strategy can be utilized to help participants solidify their understanding of the workshop topics, especially the ones in which there was difficulty.

The participants' feedback in the cognitive debriefing indicated that the curriculum should continue and be promoted to other community members. One participant said, “The leaders in Ponce are very agile and they will love this type of workshop… This is going to give them usefulness in their communities.” One participant even went on to say that the workshop was very rewarding compared with other workshops he had participated in and said (speaking of recommending the workshop to others), “It is the first time in all my years working with the community that I have received any training like this; that is, I have been in lots of workshops and they talked about many good topics but that they [the current workshops] were so precise, so clear, so specific, at least for me, it was the first time.” The participants also mentioned improvements, such as extending existent sessions or dividing the topics into more days and increasing the practical activities of each topic.

## Conclusion

The primary objective of this article is to report the development of a curriculum and evaluate its feasibility, its acceptability, and the knowledge gained by community leaders. The study findings affirm that the curriculum is feasible and acceptable. Furthermore, it supports the preliminary effectiveness of the curriculum concerning improving the knowledge of community leaders. Although other curriculums have been created to capacitate community members about community-based participatory research, health disparities, and research,^5,11,16–20^ ours is, to our knowledge, the first curriculum tailored to Hispanic community leaders that integrates various topics. This curriculum may aid in integrating community members more effectively in research activities that will support in creating meaningful studies for the communities they impact and ultimately assist in increasing health equity through community-based participatory research. The strengths of the study include utilization of a mixed methodology and obtaining a participant retention rate of 100% during the implementation period. Limitations of the study include participation of leaders, only, as they have an extra motivation to participate in this type of workshop. Furthermore, the statistical methodology may have been more rigorous if we had used paired *t*-tests to analyze the knowledge gained. Being that this was a pilot project intended to ascertain the preliminary effectiveness of the curriculum, future studies should aim to evaluate effectiveness using a randomized controlled trial to obtain generalizability of results.

## Abbreviations Used

CBPR: community-based participatory research
95% CI: 95% confidence interval
FRM: fundamentals of research methodology
HD: health disparities
MD: mean difference
SDH: social determinants of health
SED: standard error difference
TR: translational research
